# Fluid therapy should be as short as possible

**DOI:** 10.62675/2965-2774.20250310

**Published:** 2025-05-13

**Authors:** Romina Aparecida dos Santos Gomes, Alexandre Rodrigues Ferreira, Adriana Teixeira Rodrigues, Maria do Carmo Barros de Melo, Jaisson Gustavo da Fonseca

**Affiliations:** 1 Universidade Federal de Minas Gerais Faculdade de Medicina Hospital das Clínicas Belo Horizonte MG Brazil Departamento de Pediatria, Hospital das Clínicas, Faculdade de Medicina, Universidade Federal de Minas Gerais - Belo Horizonte (MG), Brazil.

## INTRODUCTION

Hemodynamic stabilization with intravenous fluids remains a major therapeutic challenge in patients with shock. Excessive fluid resuscitation can lead to fluid overload (FO), contributing to unfavorable outcomes such as increased duration of mechanical ventilation, prolonged hospital stay, need for renal replacement therapy, and increased risk of mortality.^(1,2)^

Fluid overload is defined as the accumulation of fluids above 10% of body weight^(3,4)^ and is calculated in most studies via the following formula:^(5)^


$$\text{FO}\%=\frac{(\text{administered fluids in liters}-\text{eliminated fluids in liters})\times 100}{\text{weight in kg on admission}}$$

Although this formula does not consider other components of the fluid balance, such as insensible losses, other losses, and endogenous water production, it can serve as a guide for clinical use in general situations. When these other losses are considerable, they should be added to the total eliminated fluids, summing them to the diuresis volume.

Numerous questions remain regarding the type, dose, and timing of fluid administration. Both the characteristics of the fluids and the strategy for their administration are important. Fluids should be considered medicines with specific indications, contraindications, and potential adverse effects.^(6)^

The current concept of "fluid management" focuses on four questions (when to start and stop fluid therapy and when to stop fluid removal), four indications (resuscitation, maintenance, replacement, and nutrition), and four Ds (drug, dosing, duration, and de-escalation).^(7)^

**De-escalation** refers to the beginning of the process of reducing or interrupting fluid therapy.^(7)^

**Figure 1:**
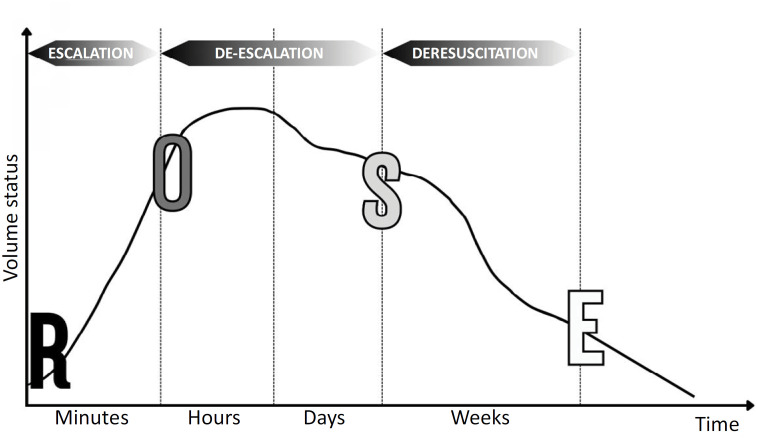
ROSE conceptual model with 4 phases of fluid therapy (resuscitation, optimization, stabilization and elimination) and 3 stages: escalation, de-escalation and deresuscitation.

Source: modified from Malbrain ML, Martin G, Ostermann M. Everything you need to know about deresuscitation. Intensive Care Med. 2022;48(12):1781-6.^(7)^

Recently, a model of fluid therapy in shock has been suggested with four distinct dynamic phases: resuscitation, optimization, stabilization, and evacuation (acronym **ROSE**) and three stages: escalation, de-escalation, and deresuscitation (Figure 1).^(7)^

**Resuscitation**: In this initial phase, usually during the first 3 to 6 hours after the start of therapy, fluid resuscitation is commonly administered according to an early, appropriate, and goal-oriented fluid management strategy. In this phase, the accumulated fluid overload (AFO) should be positive.^(8)^

**Optimization**: The second phase occurs within a few hours and involves ischemia and reperfusion. In this phase, fluid accumulation reflects the severity of the disease and can be considered a "biomarker". The greater the fluid requirement is, the sicker the patient is, and the more likely that organ failure may occur. ^(9)^ In this phase, the AFO should be neutral.

**Stabilization**: In this phase, fluid therapy is only required for ongoing maintenance, addressing normal fluid loss and replacement if the patient is experiencing ongoing loss due to unresolved pathological conditions. In this phase, the AFO should be neutral or negative.^(8)^

**Evacuation**: In this phase, the patient may recover further, entering the "flow" phase with the spontaneous elimination of excess fluids that were previously administered, or, as is the case with many critically ill patients, the patient may remain in a "nonflow" state followed by increased permeability syndrome. In this phase, the AFO should be negative.

For patients in a "nonflow" state, delayed fluid removal can be achieved through the use of conservative fluid therapy and deresuscitation strategies.^(3)^

The term **deresuscitation** is defined as the active removal of fluids in patients with FO. Deresuscitation should be considered when FO negatively impacts organ function. Measures to remove excess fluid can be pharmacological (drugs) or nonpharmacological (ultrafiltration), combined with fluid restriction. Deresuscitation should be stopped once the goal has been achieved. This goal may be related to fluid balance or to clinical and laboratory criteria.^(9)^

Recently, methods for assessing FO and deresuscitation, such as lung and inferior vena cava ultrasonography^(10)^ and bioimpedance analysis,^(11)^ which contribute to favorable clinical outcomes in critically ill patients, have shown promise in the literature. The current literature also shows that deresuscitation should be individualized, as described in a study by Ma et al.,^(12)^ who evaluated resuscitation and deresuscitation in patients with septic shock classified into five groups, which can be easily identified with routine clinical variables.

Studies suggest the active removal of fluids through the use of drugs and/or ultrafiltration and that the strategy of conservative fluid management is superior to deresuscitation. In these studies, the most frequently studied clinical outcomes were the duration of mechanical ventilation, progression to renal replacement therapy, length of stay in the intensive care unit, and mortality.^(13,14)^

We performed a retrospective study that analyzed the effects of FO on mechanical ventilation, renal replacement therapy, and progression to discharge or death in critically ill children in a pediatric intensive care unit.^(15)^ Data from 70 critically ill patients, with a mean age of 6.8 ± 6 years, were analyzed. AFO on the third day of hospitalization proved to be a determining factor in the clinical outcomes of extubation, initiation of renal replacement therapy, discharge from intensive care, and death among these children.

Therefore, we strongly encourage prospective studies that include a larger sample size and evaluate conservative fluid management protocols as well as the use of deresuscitation to control FO in critically ill patients. We suggest the collection of data to assess the interrelationship with ventilator weaning (time on mechanical ventilation, progression to mechanical support, and extubation), the use of vasopressors and/or inotropic drugs (duration, maximum dose, and discontinuation), renal replacement therapy (initiation and discontinuation), and the length of stay in intensive care and death.
